# Practical Points

**Published:** 1897-07-31

**Authors:** 


### PRACTICAL POINTS.

*Cottage Hospitals.—The Question of Site.—A correspondent writes: "I venture to write to ask whether you consider the proximity of a railway station a serious objection to a site for a cottage hospital."*

We gather from our correspondent's letter that the inmates of any cottage hospital erected on the site near the railway would be subject to the continuous noise of shunting trains in the goods department throughout the day, that is, from early morning till late in the evening; and that during the night time they would have the further disadvantage of a good many main line trains passing in the night. No doubt if the occupants of the hospital were in good health, and if they were to continuously reside there, at the end of six months or a year they would cease to be disturbed by the noises in question. But presuming that many of the cases must necessarily be accident cases, suffering from shock, and that all the inmates will be ill, we cannot fail to realise the disadvantages and the dangers which must attach to the railway site, so far as a goodly number of patients each year are concerned. We think, therefore, the gravest consideration should be given to these points before it is determined to erect a hospital upon a piece of ground subject to the disturbing noises just referred to. As there are two available sites which are identical from a hygienic and architectural point of view, we are strongly of opinion, judging from the facts mentioned, that it would be better in all the circumstances to select the quieter site, notwithstanding the fact that it is off the main road and some half a mile distant from the centre of the town.

